# Identification of a Novel Astrovirus Associated with Bovine Respiratory Disease

**DOI:** 10.1155/2023/8512021

**Published:** 2023-04-04

**Authors:** April Nelsen, David Knudsen, Ben M. Hause

**Affiliations:** ^1^Department of Veterinary and Biomedical Sciences, South Dakota State University, Brookings, South Dakota, USA; ^2^Animal Disease Research and Diagnostic Laboratory, South Dakota State University, Brookings, South Dakota, USA

## Abstract

Astroviruses (AstVs) cause gastrointestinal disease in mammals and avians. Emerging evidence suggests that some AstVs have extraintestinal tissue tropism, with AstVs detected in the liver, kidney, central nervous system, and the respiratory tract variably associated with disease. In cattle, AstV infection has been linked to gastroenteric or neurologic disease. Here, metagenomic sequencing of a lung from a bovine with respiratory disease identified a novel AstV with a predicted capsid-encoding ORF2 amino acid sequence with 66% identity to caprine astrovirus (CAstV G2.1). A quantitative reverse transcription PCR (qRT-PCR) targeting ORF2 found four out of 49 (8%) lungs and one out of 48 (2%) enteric samples obtained from bovine diagnostic submissions positive for the novel bovine astrovirus (BAstV). In two strongly qRT-PCR-positive lung samples, intense novel BAstV nucleic acid signals were mainly localized in the cytoplasm of alveolar macrophages and mononuclear cells using RNAscope® in situ hybridization (ISH). Genetic analysis of two novel BAstV genomes determined from qRT-PCR positive samples found high similarity for ORF1ab nucleotide sequence (92.1% and 93.9%) to BAstV strain BSRI-1, while ORF2 nucleotide sequence was most similar to CAstV G2.1 (74.6% and 77.6%). Phylogenetic analysis of the novel BAstV sequences found a close genetic relationship to the single BAstV (BSRI-1) previously identified from a bovine respiratory sample as well as bovine and caprine AstVs identified from various tissues. Further research is needed to determine the clinical significance of BAstV in respiratory diseases.

## 1. Introduction

Bovine astrovirus (BAstV) is a member of the *Astroviridae* family, genus *Mamastrovirus*, which includes astroviruses using mammalian hosts[1]. A large number of astrovirus species have been identified and classified based on their host, which do not correspond to genetic phylogenies [2]. The nonenveloped virion contains a positive sense, 6.4–7.7 kb, single-stranded RNA [1]. The genome contains three open reading frames (ORFs): ORF1a, ORF1b, and ORF2 [3]. A frameshift between ORF1a and ORF1b allows for expression of ORF1ab that encodes nonstructural proteins, including protease and RNA-dependent RNA polymerase [3]. ORF2, at the 3′ end of the genome, encodes the capsid protein [3].

The transmission of astroviruses (AstVs) is primarily via the fecal-oral route [4]. While a majority of AstVs have been isolated from gastrointestinal samples, AstVs are increasingly being identified in extraintestinal tissues, often associated with clinical disease, including the liver, kidney, and neurologic disease [5]. Although astroviruses have been detected in respiratory tract samples, no clear association has been made between astroviruses and respiratory diseases. In cattle, a case-control study used metagenomic sequencing to identify viruses associated with bovine respiratory disease (BRD) [6]. Four symptomatic cattle were positive for BAstV, whereas all asymptomatic cattle were negative [6]. The association between BastV detection and clinical disease did not reach statistical significance however due to the small number of positive symptomatic calves.

Bovine astrovirus was first detected in 1975 from fecal samples of British diarrheic calves and has since been identified with varying prevalence in the United Kingdom, the United States, China, Korea, Japan, Brazil, Egypt, Turkey, Switzerland, Italy, Germany, Canada, and Uruguay [1, 7–21]. Several of these countries reported BAstV detection both in fecal samples and in neurologic tissues, as well as the United States reporting BAstV in nasal swabs of cattle showing respiratory symptoms. Animals are not routinely screened for AstV, thus prevalence information is limited to surveillance studies. A recent study showed that overall prevalence of BAstV in diarrheic calves was 55.17% and 36.36% in asymptomatic calves [22]. Additionally, BAstV is more common in younger cattle compared to adult cattle [10, 23].

Other mamastroviruses have also been detected in animals with respiratory disease. One study detected a novel porcine astrovirus 4 (PAstV-4) from young pigs exhibiting unexplained acute respiratory disease. PAstV-4 RNA levels were significantly higher in nasal as opposed to fecal swabs, suggesting a possible respiratory tropism for PAstV-4 [24]. Another study detected a 25% overall prevalence of PAstV, including PAstV1, PAstV2, and PAstV5, in pig lung samples [25].

While performing routine metagenomic sequencing of diagnostic samples in our lab, a novel astrovirus was detected in a bovine lung sample submitted for testing due to respiratory disease. As limited information has been reported on astrovirus association with respiratory disease in cattle, we explored the prevalence, genetics, and pathology associated with respiratory BAstV infection.

## 2. Materials and Methods

### 2.1. Sample Selection

Retained frozen tissue samples submitted for diagnostic testing from December, 2020, to December, 2021, were randomly selected from bovine submissions that included lung tissues or enteric tissues. Lung tissue (*n* = 49) and enteric tissue (*n* = 48) were homogenized in phosphate buffered saline (PBS) and stored at −80°C. Detailed background information on the clinical samples was not consistently available and was not included in this study.

### 2.2. Quantitative Reverse-Transcription PCR

Nucleic acids were extracted from homogenized tissue with the QIAamp® Viral RNA Mini Spin or MagMax™ Viral/Pathogen Nucleic Acid Isolation Kit as per manufacturer's instructions. Quantitative reverse-transcription PCR (qRT-PCR) was performed as per the manufacturer's instructions using Fast Virus 1-step reagents (ThermoFisher). A master mix consisted of TaqMan® Fast Virus 1-step Master Mix, BAstV primers and probe, and RNase-free water was added to the sample and was run on Applied Biosystems 7500 FAST Real-Time PCR System with an initial reverse transcription step for 5 minutes at 50°C 20 seconds at 95°C followed by 40 cycles of 15 seconds at 95°C and 1 minute at 60°C. Primers (forward primer, CTTATGCAGAACCCTCAG; reverse primer, CAGCCAAGCGTTTTATCACC) and probe (56-FAM/CCACCAACC/ZEN/TCCCTTGAACAACCA/3IABkFQ/) were designed to target ORF2 from the novel BAstV (GenBank Accession ON191568).

### 2.3. Metagenomic Sequencing

Metagenomic sequencing was performed on archived frozen tissue samples that were qRT-PCR positive for the novel BAstV with a cycle threshold (Ct) value less than 25. Metagenomic sequencing was performed as previously described [26, 27].

### 2.4. Bovine Astrovirus Histopathology and In Situ Hybridization

Novel BAstV qRT-PCR-positive cases (Ct < 25) with archived formalin-fixedparaffin-embedded (FFPE) tissue blocks were selected for histopathology.

The distribution of the novel BAstV in fixed lung tissue was determined by in situ hybridization (ISH). ISH was performed using the commercial RNAscope® system (Advanced Cell Diagnostics) according to the manufacturer's instructions. The counterstain for ISH was Gill's Hematoxylin II. The proprietary novel BAstV probe (part ID #1176901-C1) was designed by ACD based on the novel BAstV ORF2 gene sequence determined here (GenBank Accession ON191568). Manufacturer-provided probes were used as controls, along with a novel BAstV qRT-PCR negative lung slide as a negative comparison control.

Hematoxylin and eosin staining of serial lung sections was performed at South Dakota State University Animal Disease Research and Diagnostic Laboratory Histology section using a standard protocol.

### 2.5. Genetic and Phylogenetic Analysis

To explore the relationship of the novel BAstV identified in respiratory tissues to previously determined mamastroviruses, reference astrovirus genomes were downloaded from GenBank. Reference genomes and protein sequences included 59 BAstVs, 73 mamastroviruses from other hosts, and 4 avastroviruses (Table S1). Phylogenetic analyses were performed on ORF1ab and ORF2 amino acid sequences using the best fitting LG + G + I + F model of evolution with 500 bootstrap replicates as implemented in the MEGA11 software program.

### 2.6. Recombination Detection

All astrovirus genomes that were downloaded from GenBank (Table S1) were used for recombination analysis. AstV genomes were aligned using MEGA 11, and recombination detection was performed using RDP4, which used RDP, GENECOV, Bootscan, MaxChi, Chimaera, SiSscan, and 3Seq algorithms for recombination detection.

## 3. Results and Discussion

A lung sample from a 7-month-old bovine from South Dakota was submitted for diagnostic testing as case 20-25551. Clinically, the calf exhibited labored breathing and nasal discharge prior to being found dead. Aerobic culture isolated *Mannheimia haemolytica* and *Pasteurella multicoida* from both lung and heart valve tissues. All other bovine respiratory pathogen testing, including bovine respiratory syncytial virus (BRSV), bovine coronavirus (BCV), *Mycoplasma bovis*, bovine viral diarrhea virus (BVDV), bovine herpesvirus-1 (BHV-1), and influenza D virus (IDV), was negative. Histopathology noted severe subacute diffuse bronchointerstitial pneumonia with septal and pleural edema and inflammation. No cardiac lesions were observed. Metagenomic sequencing identified a novel BAstV with an ORF2 gene sequence most similar to CAstV G2.1 with 74.6% identity, prompting us to investigate the prevalence and significance of this AstV in bovine respiratory disease.

### 3.1. Detection of Bovine Astrovirus by qRT-PCR

A low prevalence of the novel BAstV was detected in archived lung (4/49, 8%) and enteric tissues (1/48, 2%). Positive lung samples had cycle threshold (*C*_*t*_) values of 18.5, 24.6, 33.0, and 35.3, while the sole positive enteric tissue had a *C*_*t*_ value of 35.9. The lung homogenate from case 20-25551 had a *C*_*t*_ = 18.5, which served as the positive control for qRT-PCR. A previously sequenced lung sample, negative for novel BAstV, served as the negative control. While the prevalence was low in both tissue types, novel BAstV was more frequently detected in lung tissue, suggesting this strain of BAstV may have a tropism for the bovine respiratory tract; however, chi-square analysis of the qRT-PCR results found that they were not significantly different (*P*=0.18). The lung and enteric tissues were tested based on availability. Not testing other tissue types to evaluate tissue tropism more broadly is a limitation of this study. Additionally, other BAstV genotypes were not tested in this study. Further studies need to be conducted in order to confirm the tissue tropism and qRT-PCR sensitivity for the novel BAstV.

### 3.2. Metagenomic Sequencing and Genetic and Phylogenetic Analysis

The genomes of BAstV were determined by metagenomic sequencing of the two lung tissues with *C*_*t*_ values less than 25. The complete genome of strain 25551 (BAstV-25551) and the near-complete genome of strain 21-24401 (BAstV-21-24401) were submitted to GenBank as accessions ON191568 and ON552247, respectively. The two BAstV genomes that we determined had >98% nucleotide identity. BAstV-25551 comprised 6,127 nucleotides and had the highest sequence identity (91.9%) to BAstV isolate BSRI-1 (KP264970), which was identified by metagenomic sequencing of a nasal swab from a calf with respiratory disease [6]. The partial genome sequence of BAstV-21-24401 comprised 5,349 nucleotides and likewise was most similar to BAstV BSRI-1 with 94.06% identity. No additional viruses were identified. This contrasts with the metagenomic sequencing study of a large calf ranch that identified BAstV strain BSRI-1, where bovine adenovirus 3 (BadV-3), bovine rhinitis A virus (BRAV), bovine rhinitis B virus (BRBV), and IDV were also detected and associated with BRD [6]. The ORF1ab nucleotide sequences of BAstV-25551 and BAstV-21-24401 were 92.1% and 93.9% identical, respectively, with BSRI-1. However, the ORF2 nucleotide sequence, that encodes the capsid proteins, showed the highest sequence identity, 74.6% and 77.6%, respectively, to caprine astrovirus G2.1 (MK404645), which came from an unpublished study of novel astroviruses in small ruminants.

**Figure 1:**
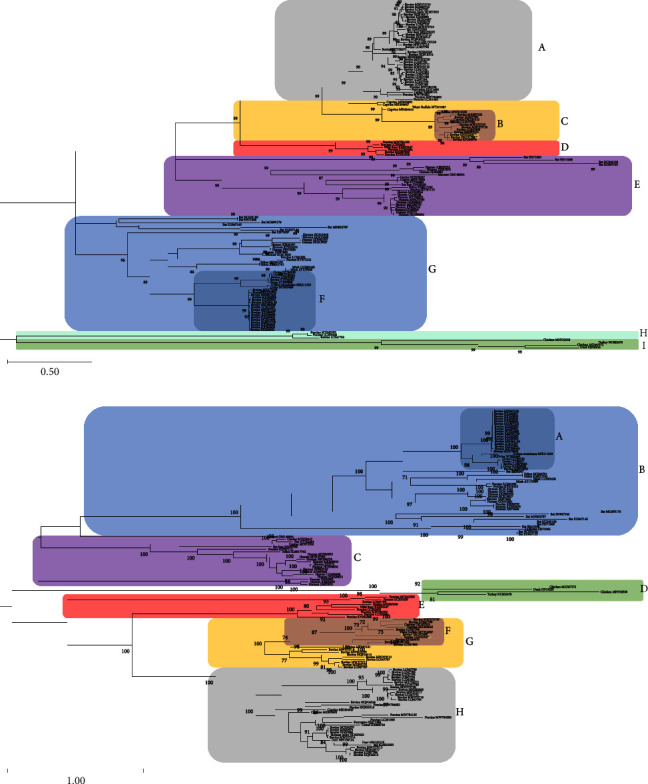
Phylogenetic analysis of astrovirus. Phylogenetic trees were constructed by maximum likelihood analysis with 500 bootstrap replicates using MEGA11 software. Bootstrap values ≥70 are shown above the branches. GenBank accession numbers are indicated along with AstV host. The scale bar indicates 0.5 substitutions per site for Figure 1(a) and 1.0 substitution per site for Figure 1(b). ORF1ab amino acid sequence clades are arbitrarily labeled A-I. Clade B contains BAstV amino acid sequences identified in this study and highlighted in yellow (a) ORF2 amino acid sequence clades are arbitrarily labeled A-H. Clade F contains BAstV amino acid sequences identified in this study highlighted in yellow (b).

Phylogenetic analyses were performed to interrogate the evolutionary history of BAstV-25551 and BAstV-21-24401. Amino acid sequences for ORF1ab formed nine distinct, well-supported lineages which were arbitrarily labeled A-I (Figure 1(a)). A majority of the sequences in lineage A were from bovines with enteric disease or without specified symptoms. BAstV-25551 and BAstV-21-24401 clustered with lineage B strains which included bovine and caprine strains. Included in lineage B was the respiratory BAstV strain BSRI-1, four caprine AstVs, one water buffalo AstV, and eight additional BAstV, two of which were identified in cattle with neurologic disease, with the remainder from enteric samples or unspecified. Lineage B sequences were most closely related to caprine and water buffalo strains in lineage C. The majority of BAstVs identified in neurologically diseased cattle clustered in clade F, where only one of the 19 BAstV sequences was identified from a bovine with enteric disease. Clade F also contained four ovine AstVs and one muskox; one of the ovine AstVs was identified in a neurological case. Clade D AstV mostly originated from pigs though it contained a single bovine strain. The remaining lineages contained AstV originating from a variety of hosts, including humans, bats, canines, and felines.

Similar to the ORF1ab, phylogenetic analysis of ORF2 amino acid sequences found clustering of strains generally related to host and clinical disease. ORF2 is the most divergent open reading frame of AstVs and a common site of recombination [28]. The capsid protein allows for receptor binding and cell infection and consequently is the target of neutralizing antibodies [29]. Capsid mutation, recombination, and cross-species transmission allow virus to escape host humoral immunity [4]. ORF2 amino acid sequences identified in this study clustered with Clade F, which also contained six other BAstVs, including strains from respiratory and neurologic tissues, as well as two caprine AstVs, and one water buffalo AstV (Figure 1(b)). Additional nine BAstVs clustered in Clade G. Similar to the phylogeny based on ORF1ab, ORF2 clade A mainly comprised BAstV isolated from cattle with neurologic disease. The majority of the remaining BAstV was found in clade H and included sequences identified in animals with enteric symptoms or no identified symptoms. Besides the 23 BAstVs in clade H, close relatives were also identified in pigs (*n* = 4), roe deer (*n* = 2), goats (*n* = 2), a porcupine, a camel, and a yak. Previous studies noted AstV interspecies transmission, with nearly identical AstV capsid proteins identified in sheep with encephalitis and cattle with neurologic disease [30]. Numerous instances of suspected interspecies transmission were evident in our phylogenetic analysis, where highly homologous clades comprised multiple strains from a single species which included a strain from a different host. Previous analysis of BAstV ORF2 amino acid sequences showed some strains clustered with porcine astrovirus type 5 and ovine astrovirus, further corroborating the findings of this study that cross-species transmission of AstVs may occur which may result in a variety of clinical signs and diverse tissue tropism [11].

### 3.3. Recombination Detection

To explore potential recombination events, reference astrovirus genomes were downloaded from GenBank (Table S1) for use in program RDP4. A total of three possible recombination events were detected in BAstV-25551 and two in BAstV-21-24401 (Table S2). Major parental sequences for possible recombination events include caprine astroviruses (MK404647 and OK107513) and bovine astroviruses (MW810339 and ON191568). Using the MaxChi analysis, a recombination event starting at position 2485 and ending at position 2893 was predicted in BAstV-25551 and BAstv-21-24401 with the major parent being MK404647 and the minor parent being Z25771. The recombination event occurred in ORF1ab and was supported by 2 out of 7 algorithms in RDP4. The second recombination event for BAstV-25551 occurred at starting breakpoint 6820 and ending breakpoint 6903 using the GENECOV analysis, further supported by 3 out of 7 programs in RDP4. The final recombination event for BAstV-25551 was predicted with the major parent OK107513 and the minor parent MK404645 with the beginning and end breakpoints of 7647 and 8145, respectively. This event was supported by 3 out of 7 programs in RDP4, and both the major and minor parent strains of this recombination event, occurring in ORF2, originated from caprine astroviruses. The final recombination event for BAstV-21-24401 occurred in ORF2 with the beginning break point of 7822, and the end breakpoint of 7914 was supported by 3 out of 7 programs in RDP4. The major parent for this event was ON191568, and the minor parent was HQ91313.

Recombination events have been observed in different regions of the genome for other AstVs, including pigs [31, 32]. These results, in addition to phylogenetic similarities to CAstV 2.1, suggest that the novel BAstV identified here originated from recombination between bovine and caprine AstVs which may explain interspecies transmission and atypical respiratory tropism leading to bovine respiratory disease.

### 3.4. Detection and Characterization of Novel BAstV Nucleic Acid by ISH in Bovine Respiratory Disease Cases

**Figure 2:**
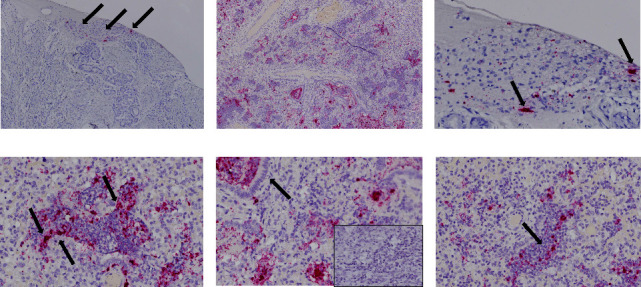
Detection of the novel bovine astrovirus nucleic acid in lungs from cattle with respiratory disease by in situ hybridization (ISH). Signal for the novel BAstV ranged from patches in 21-24401, indicated by arrows (a), to intensely diffuse in 20-25551 (b), 100x. Pinpoint signals from 21-24401 were detected in the cytoplasm of cells that corresponded to large alveolar macrophages, as well as mononuclear cells indicated by arrows (c), 400x. Diffuse, intense novel BAstV ISH signals obscured entire cells in sample 20-25551 indicated by arrows (d), 400x. No signal was detected in the bronchial epithelium, indicated by arrow, (e) or neutrophils, indicated by arrow, (f), in 20-25551 and 21-24401, respectively 400x. A negative control is inserted in (e).

**Figure 3:**
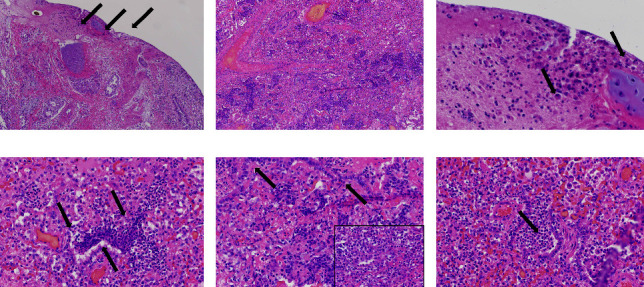
Hematoxylin and eosin staining of serial lung sections used for ISH analysis. Arrows indicate areas where the novel BAstV ISH signal was identified in patches of 21-24401 (a), 100x. Serial H&E stained section with an intense diffuse ISH novel BAstV signal of 20-25551 (b), 100x. Large alveolar macrophages and mononuclear cells are indicated by the arrows (c, d), 400x. Bronchiolar epithelial cells, indicated by arrows, (e) and neutrophils, indicated by arrow, (f), 400x. A negative control is inserted in (e).

In situ hybridization for the novel BAstV was performed on a fixed tissue from two qRT-PCR-positive lungs. Novel BAstV ISH signal distribution varied from focal (Figure 2(a)) to intensely diffuse (Figure 2(b)). The probe signal was visualized as pinpoints in the cytoplasm of cells that corresponded to alveolar macrophages and other interalveolar inflammatory cells (Figures 2(c) and 3(c)). Diffuse, intense novel BAstV ISH signals often obscured entire cells (Figures 2(d) and 3(d)). No novel BAstV ISH signal was detected in the bronchial epithelium (Figures 2(e) and 3(e)). Lung samples negative for novel BAstV showed no ISH signal (Figures 2(e) insert, 3(e) insert). Neutrophil infiltration (Figures 2(f) and 3(f)) was devoid of novel BAstV ISH signals, consistent with bacterial coinfection. Bacterial infections are thought to be opportunistic to viral infections or other agents causing primary damage to epithelium in BRD, such as mycoplasma or inhaled ammonia and/or other gases [33]. We hypothesize that respiratory BAstV infection may serve as a primary viral insult that predisposes cattle for secondary bacterial infection, though further research is needed to confirm this hypothesis.

Histopathology on case 21-24401 noted alveoli and small airways containing neutrophils or alveoli that contained necrotic cellular debris mixed with inflammatory exudate, consistent with bronchopneumonia (Figures 3(a), 3(c), and 3(f)). Case 21-24401 was an 8-month-old calf from South Dakota submitted for nonspecific neurological signs. Aerobic culture isolated *Histophilis somni* from both lung and brain tissues. Other bovine respiratory pathogen testing, including BRSV, BCV, *Pasteurella multocida*, and *Mannheimia haemolytica,* was negative by PCR. *Mycoplasma bovis* was positive by PCR. The histopathology of case 21-24401 showed neutrophils in areas that novel BAstV ISH signal was not detected (Figure 2(f)). This case identified *M. bovis*, bacteria associated with BRD, which is consistent with neutrophil infiltration. No other viral respiratory pathogens aside from novel BAstV were detected through metagenomic sequencing or PCR testing.

Histopathology on 20-25551 showed severe diffuse bronchointerstitial pneumonia and inflammation (Figures 3(b), 3(d), and 3(e)). Similar to case 21-24401, bacteria associated with BRD, *M. haemolytica* and *P. multicoida*, were detected in the absence of other viral respiratory pathogens. Novel BAstV ISH signal was detected in the alveolar macrophages of both BRD cases showing pneumonia (Figures 2(b) and 2(d)). While macrophages are well known to promote inflammation and phagocytize, they may also be the host for viral replication in some respiratory viruses, such as bovine parainfluenza 3 virus (PI3V) [34].

Despite advances in the discovery of novel astroviruses and their identification in diverse tissues, little is known about their pathogenesis [5, 20, 35]. Here, we distinctly detected novel BAstV nucleic acid in cells corresponding to alveolar macrophages and multinucleated giant cells and other interalveolar inflammatory cells. The identification of bacteria associated with BRD, but no significant BRD viral pathogens in both pneumonia cases aside from novel BAstV, suggests that novel BAstV may be a primary or secondary pathogen in bovine respiratory disease. Studies using RNAscope® ISH have been conducted to detect viruses that are difficult to isolate or lack diagnostic tools for identification and are informative on viral pathogenesis [36–38]. However, it is unclear if the ISH signal observed here in alveolar macrophages is due to phagocytosis or active replication. Controlled inoculation with a respiratory novel BAstV isolate is needed to fully investigate the tissue tropism of novel BAstV.

ISH was also performed on brain tissue for 21-24401 and heart tissue for 20-25551; however, no novel BAstV ISH signal was detected in either of those tissues.

The BAstV identified in this study shows sequence homology with BAstV BSRI-1 recovered from BRD cases, as well as caprine astrovirus G2.1. The results contribute to the emerging evidence of diverse tissue tropism and cross-species transmission of AstVs. While qRT-PCR was only performed on lung and enteric tissues, ISH was performed on all tissues for the two diagnostic cases that were strongly positive for novel BAstV and failed to detect signal in brain or cardiac tissue. Further studies on the pathogenesis of novel BAstV are needed to ascertain its tissue tropism and etiologic significance.

## Data Availability

The genome sequences for 20-25551 and 21-24401 are available at GenBank as ON191568 and ON552247, respectively.

## Supplementary Materials

Supplementary Materials Table S1: Reference Strain Information from GenBank including strain, host, clinical signs, GenBank accession number, time, and location. Table S2: Recombination detection in novel BAstV.
