# Supplementary material for: Identification of a Novel Astrovirus Associated with Bovine Respiratory Disease
Source: Transbound Emerg Dis. 2023 Apr 4;2023:8512021. doi: 10.1155/2023/8512021 (PMC12017199; doi:10.1155/2023/8512021)
Supplement: Supplementary Materials — Table S1: Reference Strain Information from GenBank including strain, host, clinical signs, GenBank accession number, time, and location. Table S2: Recombination detection in novel BAstV. [file 8512021.f1.zip › Table S2 (1).pdf]

Table S2. Recombination detection in the novel BAstV

Event #	Recombinant genome			Detection Methods						
				RDP	GENECONV	Bootscan	Maxchi	Chimaera	SiSscan	3Seq
1	**ON191568**	**Bovine astrovirus strain 25551**		NS	NS	NS	3.24E-01	NS	1.04E-02	NS
	major parent	MK404647	Caprine astrovirus G5.1							
	minor parent	Unknown (Z25771)	Human astrovirus type 1							
	note: recombination event detected by 2 of 7 programs. Flagged with multiple warnings.									
2	**ON191568**	**Bovine astrovirus strain 25551**		4.23E-02	1.76E-03	NS	NS	NS	NS	2.95E-02
	major parent	MW810339	Bovine astrovirus strain 51-Astroviridae-16							
	minor parent	MZ367372	Chicken astrovirus isolate CAV/Belgium/4134_001/2019							
	note: recombination event detected by 3 of 7 programs. Flagged as possible misidentification of true recombinat. MW810339 could be the true recombinant. Flagged with multiple other warnings.									
3	**ON191568**	**Bovine astrovirus strain 25551**		7.46E-04	NS	NS	3.61E-02	NS	4.93E-09	NS
	major parent	OK107513	Caprine astrovirus strain SWUN/F2/2019							
	minor parent	MK404645	Caprine astrovirus G2.1							
	note: recombination event detected by 3 of 7 programs. Flagged as possible misidentification of true recombinat. OK107513 or MK404645 could be the true recombinant. Flagged with multiple other warnings.									
4	**ON552247**	**Bovine astrovirus strain 21-24401**		NS	NS	NS	3.24E-01	NS	1.04E-02	NS
	major parent	MK404647	Caprine astrovirus G5.1							
	minor parent	Unknown (Z25771)	Human astrovirus type 1							
	note: recombination event detected by 2 of 7 programs. Flagged with multiple warnings.									

**5**	**ON552247**	**Bovine astrovirus strain 21-24401**		5.9E10-12	1.0E10-02	NS	8.7E10-7	NS	NS	NS
	major parent	ON191568	Bovine astrovirus strain 25551							
	minor parent	Unknown (HQ91313)	Bovine astrovirus B18/HK							
	note: recombination event detected by 3 of 7 programs. Flagged with multiple warnings.									

Minor Parent = Parent contributing the smaller fraction of sequence.

Major Parent = Parent contributing the larger fraction of sequence.

Unknown = Only one parent and a recombinant need be in the alignment for a recombination event to be detectable.

NS = No significant P-value was recorded for this recombination event using the particular method in question.
