# Supplementary material for: Identification of a Novel Astrovirus Associated with Bovine Respiratory Disease
Source: Transbound Emerg Dis. 2023 Apr 4;2023:8512021. doi: 10.1155/2023/8512021 (PMC12017199; doi:10.1155/2023/8512021)
Supplement: Supplementary Materials — Table S1: Reference Strain Information from GenBank including strain, host, clinical signs, GenBank accession number, time, and location. Table S2: Recombination detection in novel BAstV. [file 8512021.f1.zip › Tabls S1.pdf]

Table S1 Reference Strain Information from GenBank

Strain	HOST	CLINICAL SIGNS	GENBANK ASCESSION NO.	TIME	LOCATION
**Bovine Astrovirus**	Bovine	Respiratory	ON191568	2020	USA
**Bovine Astrovirus**	Bovine	Respiratory	ON552247	2021	USA
**Bovine astrovirus B18/HK**	Bovine	None	HQ916313	2011	China
**Bovine astrovirus B170/HK**	Bovine	None	HQ916314	2011	China
**Bovine astrovirus B34/HK**	Bovine	None	HQ916315	2011	China
**Bovine astrovirus B76/HK**	Bovine	None	HQ916316	2011	China
**Bovine astrovirus B76/HK**	Bovine	None	HQ916317	2011	China
**BoAstV/JPN/Ishikawa24-6/2013**	Bovine	None	LC047787	2013	Japan
**BoAstV/JPN/Ishikawa9728/2013**	Bovine	None	LC047788	2013	Japan
**BoAstV/JPN/Hokkaido11-7/2009**	Bovine	Diarrhea	LC047789	2009	Japan
**BoAstV/JPN/Hokkaido11-55/2009**	Bovine	Diarrhea	LC047790	2009	Japan
**BoAstV/JPN/Hokkaido12-7/2009**	Bovine	Diarrhea	LC047791	2009	Japan
**BoAstV/JPN/Hokkaido12-18/2009**	Bovine	Diarrhea	LC047792	2009	Japan
**BoAstV/JPN/Hokkaido12-25/2009**	Bovine	Diarrhea	LC047793	2009	Japan
**BoAstV/JPN/Hokkaido12-27/2009**	Bovine	Diarrhea	LC047794	2009	Japan
**BoAstV/JPN/Kagoshima1-2/2014**	Bovine	None	LC047795	2014	Japan
**BoAstV/JPN/Kagoshima1-2/2014**	Bovine	Diarrhea	LC047796	2014	Japan
**BoAstV/JPN/Kagoshima2-3-1/2015**	Bovine	Diarrhea	LC047797	2015	Japan
**BoAstV/JPN/Kagoshima2-3-2/2015**	Bovine	Diarrhea	LC047798	2015	Japan
**BoAstV/JPN/Kagoshima2-24/2015**	Bovine	Diarrhea	LC047799	2015	Japan
**BoAstV/JPN/Kagoshima2-38/2015**	Bovine	Diarrhea	LC047800	2015	Japan
**BoAstV/JPN/Kagoshima2-52/2015**	Bovine	Diarrhea	LC047801	2015	Japan
**BoAstV/JPN/KagoshimaSR28-462/2016**	Bovine	Diarrhea	LC341267	2016	Japan
**Bovine astrovirus isolate NeuroS1**	Bovine	Encephalitis	KF233994	2011	USA
**Bovine astrovirus isolate BSRI-1**	Bovine	Respiratory	KP264970	2013	USA
**Bovine astrovirus**	Bovine	Encephalitis	MN464146	2019	Italy
**BoAstV/LVMS2704**	Bovine	Unknown	MN200263	2016	Uruguay
**BoAstV-Neuro-Uy**	Bovine	Encephalitis	MK386569	2018	Uruguay
**Bovine astrovirus CH13**	Bovine	Encephalitis	KM035759	2012	Switzerland
**Bovine astrovirus CH13/NeuroS1 isolate 23871**	Bovine	Encephalitis	KX266901	2015	Switzerland

**Bovine astrovirus CH13/NeuroS1 isolate 26730**	Bovine	Encephalitis	KX266902	2015	Switzerland
**Bovine astrovirus CH13/NeuroS1 isolate 26875**	Bovine	Encephalitis	KX266903	2015	Switzerland
**Bovine astrovirus CH13/NeuroS1 isolate 36716**	Bovine	Encephalitis	KX266904	2015	Switzerland
**Bovine astrovirus CH13/NeuroS1 isolate 23985**	Bovine	Encephalitis	KX266905	2015	Switzerland
**Bovine astrovirus CH13/NeuroS1 isolate 42799**	Bovine	Encephalitis	KX266906	2015	Switzerland
**Bovine astrovirus CH13/NeuroS1 isolate 43661**	Bovine	Encephalitis	KX266907	2015	Switzerland
**Bovine astrovirus CH13/NeuroS1 isolate 43660**	Bovine	Encephalitis	KX266908	2015	Switzerland
**Bovine astrovirus isolate CH15**	Bovine	Encephalitis	KT956903	2015	Switzerland
**BoAstV-VC34/338**	Bovine	Encephalitis	MK987099	2016	Switzerland
**BoAstV-VC34/346**	Bovine	Encephalitis	MK987100	2016	Switzerland
**BoAstV-VC34/375**	Bovine	Encephalitis	MK987101	2016	Switzerland
**BoAstV-VC65/693**	Bovine	Encephalitis	MK987102	2016	Switzerland
**BoAstV-VC65/698**	Bovine	Encephalitis	MK987103	2016	Switzerland
**Bovine astrovirus BH89/14**	Bovine	Encephalitis	LN879482	2014	Germany
**Boine astrovirus CHN/Hebei-1/2019**	Bovine	None	MW373712	2019	China
**Bovine astrovirus/CHN/Hunan-1/2019**	Bovine	Diarrhea	MW373713	2019	China
**Bovine astrovirus/ CHN/HLJ-2/2019**	Bovine	Diarrhea	MW373714	2019	China
**Bovine astrovirus/CHN/HLJ-1/2019**	Bovine	Diarrhea	MW373715	2019	China
**Bovine astrovirus/CHN/SD-1/2019**	Bovine	None	MW373716	2019	China
**Bovine astrovirus/CHN/Hubei-1/2019**	Bovine	Diarrhea	MW373717	2019	China
**Bovine astrovirus CHN/Henan-2/2019**	Bovine	None	MW373718	2019	China
**Bovine astrovirus/CHN/Henan-1/2019**	Bovine	None	MW373719	2019	China
**Bovine astrovirus/CHN/JL-1/2019**	Bovine	Diarrhea	MW373720	2019	China
**Bovine astrovirus strain 51-Astroviridae-16**	Bovine	Unknown	MW810339	2018	China
**Bovine astrovirus strain BAstV-GX7/CHN/2014**	Bovine	Unknown	NC024297	2014	China
**Bovine astrovirus CH13**	Bovine	Encephalitis	NC024498	2102	Switzerland
**BoAstV-GX7/CHN/2014**	Bovine	Diarrhea	KJ620979	2104	China
**BoAstV-GX27/CHN/2014**	Bovine	Diarrhea	KJ620980	2014	China
**Bovine astrovirus isolate BoAstV/CN/HB-SJZ/2021**	Bovine	Diarrhea	MZ603733	2021	China
**Bovine astrovirus isolate Egy-1**	Bovine	Diarrhea	MT758371	2015	Egypt
**Bovine astrovirus strain 20B05**	Bovine	Encephalitis	MZ475060	2020	South Korea
**Caprine astrovirus strain SWUN/F2/2019**	Caprine	Unknown	OK107513	2020	China
**Caprine astrovirus G2.1**	Caprine	Unknown	MK404645	2017	Switzerland
**Caprine astrovirus G5.1**	Caprine	Unknown	MK404647	2017	Switzerland

**Caprine astrovirus isolate China/ SWUN/F4/2019**	Caprine	Unknown	MZ005893	2019	China
**Water buffalo astrovirus strain BufAstV/CN/NNA17**	Water	Diarrhea	MT521687	2019	China
**AstV UK/2014/lamb**	Ovine	Unknown	LT706530	2014	UK
**OAstV/UK/2013/ewe/lib01454**	Ovine	Unknown	LT706531	2013	UK
**Ovine astrovirus**	Ovine	Unknown	NC002469	Unknown	Unknown
**OASTV-CH16 MASTV13**	Ovine	Encephalitis	KY859988	2006	Switzerland
**PoAstV-2/JPN/Bu5-10-1/2014**	Porcine	None	LC201585	2014	Japan
**PoAstV-3/JPN/Bu2-5/2014**	Porcine	None	LC201595	2014	Japan
**PoAstV-4/JPN/Bu5-10-2/2014**	Porcine	None	LC201603	2014	Japan
**PoAstV4/JPN/Buta17/2014**	Porcine	None	LC201604	2014	Japan
**PoAstV-5/JPN/Ishi-Im1-2/2015**	Porcine	None	LC201620	2015	Japan
**Mamastrovirus 3 isolate PAstV-GX1**	Porcine	Unknown	KF787112	2013	Guangxi
**Mamastrovirus 4 isolate K456**	Porcine	Unknown	KY933398	2012	Kenya
**Xinjiang mamastrovirus 6 isolate 227-342448**	Porcine	Unknown	MW784082	Unknown	China
**Xinjiang mamastrovirus 7 isolate 227-67505**	Porcine	Unknown	MW784083	Unknown	China
**Sichuan mamastrovirus 12 isolate R67-15434**	Porcine	Unknown	MW784102	Unknown	China
**Sichuan mamastrovirus 11 isolate R69-151373**	Porcine	Unknown	MW784105	Unknown	China
**Porcine astrovirus 5 strain PAstV-AH29-2014**	Porcine	Swine Fever	MT642595	2014	China
**Porcine astrovirus 3 strain NI-Brain/386-2015/HUN**	Porcine	Encephalitis	KY073232	2015	Hungry
**Mamastrovirus 3 strain WBAstV/CH/2015**	Wild boar	Diarrhea	KX033447	2015	China
**Takin astrovirus**	Sichuan takin	None	NC037655	2013	China
**Canine astrovirus strain CHN/2017/44**	Canine	Diarrhea	MF973500	2017	China
**Canine astrovirus isolate MN1-USA/ORF/2017**	Canine	Unknown	MT078247	2017	USA
**Feline astrovirus D1**	Feline	None	KM017741	2013	USA
**Mamastrovirus 2 isolate FAstV-D2**	Feline	None	KM017742	2013	USA
**Feline astrovirus D1 isolate FAstV-D1**	Feline	None	NC024701	2013	USA
**California sea lion astrovirus**	Sea lion	None	JN420358	2010	USA
**MOxAstV-CH18 MAstV13**	Ovibos	Unknown	MK211323	1982	Switzerland
**Porcupine astrovirus Hb/LP084/Guangxi**	Porcupine	Unknown	KJ571486	2011	China
**CcAstV/roe_deer/SLO/D5-14/2014**	Roe deer	None	MN150124	2014	Slovenia
**CcAstV/roe_deer/SLO/D12-14/2014**	Roe deer	None	MN150125	2014	Slovenia
**Human Astrovirus VA1/HMO-C**	Human	None	KJ920197	2014	UK
**Mamastrovirus 1 isolate kor85**	Human	Diarrhea	KP862744	2014	South Korea
**Mamastrovirus 8 isolate NI-295**	Human	Diarrhea	GQ415660	2007	Nigeria

**Astrovirus VA1**	Human	None	KY933670	2017	USA
**Human astrovirus type 1**	Human	Unknown	Z25771	Unknown	UK
**Human astrovirus 1 strain Hu/Nyergesufjalu/HUN4520/2010/HUN**	Human	Diarrhea	HQ398856	2010	Hungary
**Human astrovirus 2 strain Hu/US/2014/CA-RGDS-1072**	Human	Unknown	MN433705	2014	USA
**Human astrovirus 3 isolate 17W1028**	Human	Unknown	MK296753	2018	Ireland
**Human astrovirus 4 strain Hu/BRA/TO-207/2014**	Human	Diarrhea	MT906853	2014	Brazil
**Human astrovirus 5 isolate Goiania/GO/12/94/Brazil**	Human	Diarrhea	DQ028633	1994	Brazil
**Human astrovirus 6 isolate 192-BJ07-CHN**	Human	Diarrhea	GQ495608	2007	China
**Human astrovirus 7 strain Oxford**	Human	Unknown	MK059955	Unknown	USA
**Human astrovirus type 8**	Human	Unknown	AF260508	Unknown	Mexico
**Astrovirus VA1**	Human	Diarrhea	FJ973620	2008	USA
**Astrovirus VA2 isolate VA2/human/Stl/WD0680/2009**	Human	Diarrhea	GQ502193	2009	USA
**Astrovirus VA4 isolate VA4/human/Nepal/s5363**	Human	Diarrhea	NC019027	2008	Nepal
**Astrovirus MLB1 strain WD0016**	Human	Diarrhea	FJ402983	2008	USA
**Astrovirus MLB2**	Human	Diarrhea	AB829252	2005	Turkey
**Astrovirus VA3 isolate VA3/human/Vellore/28054/2005**	Human	Diarrhea	JX857868	2005	India
**Astrovirus MLB3 isolate MLB3/human/Vellore/26564/2004**	Human	Diarrhea	JX857870	2004	India
**HMO Astrovirus A**	Human	Diarrhea	NC013443	2007	Nigeria
**HMO Astrovirus B isolate NI-196**	Human	Diarrhea	GQ415661	2007	Nigeria
**Dromedary astrovirus**	Camelus	Unknown	KR868724	2013	United Arab
**Bat-AsV/P02**	Bat	None	MG693176	2013	Cameroon
**Mamastrovirus 14 isolate AFCD57**	Bat	None	EU847144	2005	Hong Kong
**Mamastrovirus 16 isolate AFCD11**	Bat	None	EU847145	2005	Hong Kong
**Mamastrovirus 18 isolate AFCD337**	Bat	None	EU847155	2006	Hong Kong
**Bat astrovirus Tm/Guangxi/LD77/2007**	Bat	None	NC043100	2007	Guangxi
**Mamastrovirus 18 isolate AFCD337**	Bat	None	NC043102	2006	Hong Kong
**Bat astrovirus Tm/Guangxi/LD38/2007**	Bat	None	FJ571065	2007	Guangxi
**Bat astrovirus Tm/Guangxi/LD77/2007**	Bat	None	FJ571066	2007	Guangxi
**Bat astrovirus TM/Guangxi/LD71/2007**	Bat	None	FJ571067	2007	Guangxi
**Bat astrovirus Ha/Guangxi/LS11/2007**	Bat	None	FJ571068	2007	Guangxi
**Bat astrovirus isolate BtAstB/13585-58/M.dau/DK/2014**	Bat	None	MN832787	2014	Denmark
**Mink astrovirus**	Mink	None	AY179509	2002	Unknown
**Mink astrovirus isolate SMS-AstV**	Mink	Shaking mink	GU985458	2000	Sweeden

**Yak astrovirus isolate 58**	Yak	Diarrhea	KM822593	2013	China
**Mamastrovirus 5 strain Crab-eating_fox/2016/BRA**	Cerdocyon	Central nervous	KY765684	2015	Brazil
**Marmot astrovirus strain MCAsV/XJ4/CHN/2016**	Marmot	Unknown	OM140654	2016	China
**DuAstV-1_DA06_CHN**	Duck	Unknown	FJ919225	2016	China
**Turkey astrovirus**	Turkey	Unknown	NC002470	1997	USA
**Chicken astrovirus isolate CAV/Belgium/4134_001/2019**	Gallus gallus	Respiratory	MZ367372	2019	Belgium
**Avian nephritis virus strain ANV/CHN/BJCP10-2/2018**	Gallus gallus	Unknown	MN732558	2018	China
